# Parents or Peers? (In)congruence Effect of Adolescents’ Attachment to Parents and Peers on Self-Esteem

**DOI:** 10.5964/ejop.7355

**Published:** 2023-05-31

**Authors:** Rotumba A. I. C. Karunarathne

**Affiliations:** 1 Department of Human Resource Management, Faculty of Commerce and Management Studies, University of Kelaniya, Dalugama, Sri Lanka; Maria Grzegorzewska University, Warsaw, Poland

**Keywords:** adolescents, attachment theory, parental attachment, peer attachment, self-esteem

## Abstract

Building on the attachment theory and extending prior research that has hinted strongly at the important influence of social relationships on self-esteem, this study examined the simultaneous effect of adolescents’ attachment to parents and peers on self-esteem. To test our hypotheses, we collected data from a sample of 267 adolescents. We used polynomial regression coupled with response surface analysis to assess the (in)congruence effect of adolescents’ attachment to parents and peers on self-esteem. The results of polynomial regression analysis show that the congruence effect of attachment to parents and peers did not relate to adolescent self-esteem. However, self-esteem is high when attachment to both parents and peers is at a high level rather than a low level. Moreover, results show that attachment to parents is more significant than attachment to peers in developing adolescents' self-esteem. Interpretation of findings and theoretical contribution of congruence perspective to attachment theory and self-esteem literature are discussed.

Previous research on psychology has demonstrated that social relationships play a vital role in shaping an individual’s self-esteem (e.g., Harris & Orth, 2020; Leary & Baumeister, 2000) as interactions with others help individuals to develop their social skills and emotional balance. Defined as “an individual’s subjective evaluation of her or his worth as a person” (Trzesniewski et al., 2013; p. 60), the concept of self-esteem has been investigated closely to understand the socio-emotional development of an individual (Krauss et al., 2020).

In a recent meta-analysis, Orth et al. (2018) emphasized that self-esteem developed across the lifespan and peaked at the age of 60. In the early stages of their lives, children’s social interactions are mainly limited to those with their parents. A close attachment and secure relationship with the parents are strongly correlated with greater psychological development and more positive attitude of the children (Krauss et al., 2020; Leary & Baumeister, 2000). However, as the children grow up, they develop quality relationships with their peers, which are external to their family connectedness (Gorrese & Ruggieri, 2013). Adolescents then begin to receive more feedback and assistance from their peers than from parents. Thus, there is no doubt that interactions with peers can affect the development of self-esteem among adolescents. Empirical findings suggest that the sense of self-worth of adolescents is significantly influenced by their attachment to parents (Gorrese & Ruggieri, 2013) and their attachments to peers (for more review, see a meta-analysis by Gorrese & Ruggieri, 2013). Although a substantial volume of literature has shown that attachment to both parents and peers forms the basis of adolescent self-esteem development, these researchers focused on findings of the individual association between attachment to parents and peers and self-esteem (e.g., the relationship between attachment to parents and self-esteem) and significantly ignored the joint or simultaneous effect (congruence) of parents’ and peers’ influence in shaping the adolescents’ self-esteem. Moreover, prior research has shown that adolescents’ attachment to parents is more strongly related to self-esteem development than their attachment to peers (Gorrese & Ruggieri, 2013). However, these studies have not assessed the joint effect due to attachment to parents and peers. Hence, the results of these studies do not provide sound statistical evidence to prove that the parents’ attachment is more vital than peer attachment in shaping adolescents’ self-esteem. Therefore, the present study aims to address the question of what is the simultaneous effect of adolescents’ attachment to parents and peers on the self-esteem.

## Contribution of the Present Study

This study contributes to the past research in three ways. First, although past research has repeatedly examined the role of parents and peers in building adolescents’ self-esteem, those studies examined the effect of adolescents’ attachment to parents and peers on self-esteem separately. Analysis of the simultaneous effect of attachment to parents and peers on self-esteem helps us to better understand the role of social interactions (in particular, that of parents and peers) on an adolescent’s psychological development. Thus, in this study, we attempt to specify clearly the (in)congruence effect of adolescents’ attachment to parents and peers on self-esteem. Second, by testing the combined impact of adolescents’ attachment to parents and peers on their self-esteem, this study contributes towards increasing the current understanding of attachment theory (Bowlby, 1969), particularly adult attachment. Third, we contribute to the existing literature by examining the association between attachment to parents and peers and the self-esteem of adolescents in non-Western countries. As stated by Orth et al. (2018), only a limited number of researchers have examined adolescent self-esteem outside of the Western countries; thus, knowledge about the social relationships and self-esteem of non-Western adolescents is limited. The age at which adolescents start being independent of their parents depends on the culture of a particular geographic region. Usually, Western adolescents start living away from their parents at the age of 18 (Whiteman et al., 2011). However, in South Asia, the extended family concept still prevails, and most adolescents live with their parents. Thus, the Western understanding of parental and peer attachment and self-esteem might be different from that of culturally and economically different countries. Therefore, by collecting data from a sample of adolescents in a non-western economy and analyzing it, this study hopes to contribute toward increasing our understanding of social connection and self-esteem, as it prevails in non-Western economies.

## Literature Review

### Self-Esteem

Self-esteem is considered to be one of the most influential predictors of human behavior (Harris & Orth, 2020). The earliest conceptualization of self-esteem was developed by James (1890), who considered self-esteem “to be the ratio of successes compared to failures in areas of life that are important to a given individual” (Sowislo & Orth, 2013; p. 4). James’s conceptualization of self-esteem is mainly considered the individual process of developing self-worth (Brown, 1998). Later, other researchers, such as Leary & Baumeister (2000) established another perspective of self-esteem based on the premise that interactions with others in a social network are an important predictor of one's worth as a person. Overall, the notion of self-esteem was built on self-judgment and favorable comparison with others, irrespective of how others see the person (Orth et al., 2018).

Based on how individuals respond to the available information, scholars such as Baumeister (1993) and Greenier et al. (1995) characterized self-esteem into two categories, as high self-esteem and low self-esteem. High self-esteem individuals have positive feelings about self, whereas low self-esteem individuals are uncertain or have negative feelings about self (Zeigler-Hill et al., 2013). Although the current state of knowledge on high and low self-esteem and its outcomes is inconsistent (Cameron & Granger, 2019), most researchers (e.g., Marshall et al., 2015; Orth, Robbins, & Meier., 2009) reported a negative association between low self-esteem and satisfactory outcomes such as mental well-being and connectedness to the social support networks.

Scholars examined mainly two trajectories of self-esteem, i.e., self-esteem as an outcome and as a predictor variable of objective outcomes. For instance, social relationships (Harris et al., 2017; Harris & Orth, 2020), work environment, and employee disposition (Bowling et al., 2010) were found to be determinants of self-esteem. In a meta-analysis, Bowling et al. (2010) found that self-esteem is related to organizational level outcomes, such as job satisfaction, organizational commitment, employee health, job performance, and organizational citizenship behavior. In addition, depression and anxiety (Orth, Robins, & Meier, 2009; Sowislo & Orth, 2013) and life satisfaction (Coffey & Warren, 2020) were shown to be individual-level outcomes. Besides that, prior research also discussed such ideas as global self-esteem and domain-specific self-esteem. Global evaluation of self-esteem focuses on outcomes at the global level, and domain-specific self-esteem focuses on specific level outcomes (Sowislo & Orth, 2013). In this study, we focus on the global level of self-esteem.

### Attachment Theory

Attachment theory was initially developed by Bowlby (1969) to explain the different patterns of interpersonal relationships; in particular, this theory formed the foundation of child and development psychology (Ainsworth et al., 1978). Attachment refers to the emotional bond between two individuals that remain over time (Ainsworth et al., 1978). Bowlby’s (1969, 1973) conceptualization of attachment is based upon “proximity seeking”, where individuals seek proximity or closeness (like in the case of an infant) to significant others (such as an attachment figure like a mother or caregiver) when they need protection, affection, and security (Shaver & Mikulincer, 2002). Later, some researchers (e.g., Ainsworth et al., 1978; Hazan & Shaver, 1987; Shaver et al.,1988) extended the theory to understand the adult's emotional attachment to relationship partners. Ainsworth et al.’s (1978) conceptualization of attachment includes two orthogonal dimensions, namely, anxiety and avoidance. The anxiety dimension indicates the degree to which individuals worry about rejection or about not being available to the relationship partner in need. These individuals have a fear of losing their relationship partners. In contrast, the avoidance dimension indicates that individuals feel uncomfortable and have a less emotional engagement with their relationship partners. Thus, they are self-reliant and independent (Mikulincer & Shaver, 2012). In their seminal work, Armsden and Greenberg (1987) conceptualized attachment to parents and peers in terms of three dimensions, viz. trust (trusting and respecting the needs and desires of parents and peers), communication (quality of involvement and verbal communication with parents and peers), and alienation (feelings of isolation and anger). Based on these three dimensions, Armsden and Greenberg (1987) developed a scale to assess attachment relationships, i.e., Inventory of Parents and Peer Attachment (IPPA), which is the most widely used scale to assess the adolescent’s perception towards their attachment to parents and peers.

**Figure 1:**
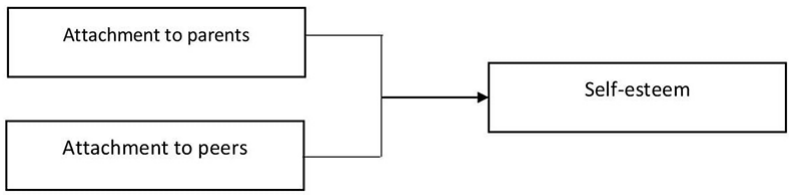
Conceptual Framework

Based on the attachment theory (Ainsworth et al., 1978; Bowlby, 1969) and prior literature (e.g., Delgado et al., 2022; Schneider et al., 2001), we developed a conceptual model. In this model, we proposed that adolescents’ attachment to their parents and peers is jointly influenced the development of self-esteem in adolescents. More specifically, we proposed that the congruence effect of adolescents’ attachment to their parents and peers would significantly influence their self-esteem. Self-esteem is high when attachment of both parents and peers is at a high level. Further, we proposed that the adolescents’ attachment to parents is more significant than their attachment to peers. The conceptual framework for this is shown in Figure 1.

### Hypotheses Development

#### Congruence Effect of Adolescents’ Attachments to Parents and Peers on Self-Esteem

Based on the attachment theory (Ainsworth et al., 1978; Bowlby, 1969; Hazan & Shaver, 1987), we first posit that congruence of attachment to parents and peers (e.g., when attachment of both parties is at a high level) will result in higher self-esteem.

Attachment theory states that adults are capable of forming emotional bonds in a variety of relationships, such as with friends and romantic partners (Shaver & Fraley, 2008). Moreover, the theory proposed that people of all ages seek proximity to others who are supportive. Young adults especially seek proximity to parents and peers in times of need (Gorrese & Ruggieri, 2013).

However, unlike in the Western countries, the children in South Asian countries typically live with their parents until they marry and thus, they have strong emotional ties with their parents. As such, we believe that even during their adolescent life, they receive emotional, informational, and instrumental support from their parents. This will eventually help them to form a positive internal model and to develop self-worth. In contrast, when the parents are not readily available at a time of need, they tend to feel insecure and may consider themselves unworthy. These ideas are aligned with previous studies that found a significant association between adolescents’ attachment to parents and their self-esteem. For instance, previous researchers (e.g., Harris et al., 2017; Mattanah et al., 2011; Orth et al., 2018) found a significant and positive association between attachment to parents and self-esteem of adolescents.

Conversely, changes may occur to individual attachment when they turn into adolescents, as individuals look for autonomy when they become older (Gorrese & Ruggieri, 2013). Consequently, adolescents tend to develop a stronger relationship with peers when their family relationships become more tenuous (Meeus et al., 2005). This kind of change becomes increasingly important for the adolescents’ psycho-emotional development (Thomaes et al., 2010). Adolescents’ interaction with peers helps them to get necessary support and interpersonal feedback (Mikulincer & Shaver, 2005). As such, peer acceptance is an important determinant of authentic self-esteem. This has been reported by scholars (e.g., Armsden & Greenberg, 1987; Gorrese & Ruggieri, 2013) who observed that a relationship bond with peers is directly related to self-esteem.

However, in real life, people interact with different parties, such as parents and peers, simultaneously. We believe that as with attachment to parents and peers is presumably a significant determinant of an adolescent's self-esteem development. This leads us to the following hypothesis:

H1: The congruence between adolescents’ attachment to parents and peers is significantly associated with self-esteem.

### Effect of Adolescents’ Attachment to Parents and Peers on Self-Esteem

The core tenet of attachment theory is that emotional connections with relationship partners are directly related to self-esteem (Harris & Orth, 2020). Attachment theory emphasizes that secure attachment is the most significant determinant of self-esteem (Mikulincer & Shaver, 2005). Securely attached individuals tend to cope positively with distress situations (Shaver & Mikulincer, 2002) and manifest joy, happiness, intimacy, affection, and sociability with relationship partners (Gorrese & Ruggieri, 2013; Mikulincer & Shaver, 2005). In contrast, insecurely attached individuals do not have strong positive relationships with their relationship partners as they have a negatively biased attitude towards relationships as well as partners (Shaver & Mikulincer, 2002). Thus, we believe that adolescents’ self-esteem is high when their attachment to parents and peers is at a high level. This leads us to Hypothesis 2:

H2: Self-esteem of adolescents is high when attachment to parents and peers is at a high-high level than when attachment to parents and peers is at a low-low level.

### Incongruence Effect of Adolescents’ Attachment to Parents and Peers on Self-Esteem

Although we proposed that the congruence effect of adolescents’ attachment to parents and peers leads to high self-esteem, we postulate that attachment to parents is more significant than attachment to peers in developing one's self-esteem. In fact, enduring emotional ties with parents is the main contribution of developing a positive internal working model, especially for children and adolescents (Gorrese & Ruggieri, 2013; Mattanah et al., 2011; Orth, Robbins, & Meier., 2009; Orth, Robbins, Trzesniewski, et al., 2009). Moreover, prior research has consistently reported a significant positive association between attachment bonds with parents and self-esteem (e.g., Bowlby, 1982; Armsden & Greenberg, 1987; Krauss et al., 2020; Orth et al., 2018). However, the empirical evidence on the connection between attachment to peers and self-esteem is inconsistent. For instance, some scholars (e.g., Armsden & Greenberg, 1987; Gorrese & Ruggieri, 2013) found a significant and positive relationship between peer attachment and self-esteem. In contrast to this, other researchers (e.g., Marshall et al., 2015; Wilkinson, 2004) found no association between peer attachment and self-esteem.

When children become adolescents, they begin to seek personal autonomy and value the company of peers. Adolescents seek feedback and support from their peers, which will eventually help them to develop self-esteem. Adolescents seek support from their peers for day-to-day concerns, such as education and fashion (Freeman & Brown, 2001). However, the emotional bond with parents plays a more crucial role in an individual’s psychological development. Support from the parents is needed when adolescents experience personal issues, fear, loneliness, loss or illness of close others, and for their long-term plans (de Jonge et al., 2022; Freeman & Brown, 2001). Especially adolescents’ attachment to parents is more significant in times of distress. Thus, we believe that adolescents’ attachment to their parents is prominent in their self-esteem development. In support to this argument, in their study, Gorrese and Ruggieri (2013) have clearly stated that attachment to parents is more important than peer attachment in shaping adolescents’ self-esteem.

Thus, we hypothesize that:

H3: Self-esteem of adolescents is higher when attachment to parents is greater than attachment to peers.

## Method

### Participants and Procedure

In line with the definition given by Sawyer et al. (2018), in this study, we defined adolescents as individuals aged from 10 to 24 years. However, our sample represented adolescents ranging in age from 19 to 24 years (late adolescence). Thus, we collected data from 267 bachelor’s students of a Management faculty in a state university of Sri Lanka. These students fell in the age group of 19–24. Therefore, we believe that our sample provides an ideal testing ground to test the hypotheses. Moreover, before doing the main study, we have done a short pilot study using 40 bachelors’ students. We collected data using an online survey and participation was voluntary. We guaranteed the confidentiality of all information provided by the students. Moreover, we sent out a cover letter along with the survey to explain the purpose of this study. In all, we sent 480 survey invitations. However, seven invitations were returned due to incorrect e-mail addresses. We received 268 questionnaires, of which we rejected one questionnaire due to incompleteness. Thus, the final sample was comprised of *N* = 267 students, which corresponds to a response rate of 55.6%. In terms of demographics, the sample was comprised of 78.3% females. Among the respondents 90.3% had at least one sibling (*M* = 1.57, *SD* = 0.94).

### Measures

Unless otherwise mentioned, all the variables were measured using a five-point Likert scale (1 = strongly disagree, 5 = strongly agree, and for reverse coded items, 5 = strongly disagree, 1 = strongly agree).

#### Self-Esteem

To measure adolescents’ self-esteem, we used the scale developed by Rosenberg (1965), which is the most widely used scale for measuring adolescents’ self-esteem. The scale comprised of 10 items which provided score on a single factor. As an example, one item is “On the whole, I am satisfied with myself”. Cronbach’s alpha for the composite was 0.89.

#### Attachment to Parents

Attachment to parents and peers was assessed using the global Inventory of Parents and Peer Attachment (IPPA) scale, which was developed by Greenberg et al. (1983) and revised by Armsden and Greenberg (1987) to assess the adolescent’s perception of their attachment to parents and peers. Although, the original scale comprised 28 items, the revised scale includes just 25 items. The instrument evaluated 3 factors, i.e., trust, communication, and alienation. Trust comprised 10 items and an example item is, “my parents respect my feelings”. Communication comprised 9 items and an example item is “my parents can tell when I am upset about something”. Alienation was measured using 6 items, and an example item is “I feel angry with my parents”. Cronbach’s alpha for the composite factors were, trust = 0.87, communication = 0.88, and alienation = 0.86. Composite reliability of the overall scale was 0.93.

#### Attachment to Peers

We measured attachment to peers using the revised version of the Inventory of Parents and Peers Attachment (IPPA) scale (Armsden & Greenberg, 1987). The instrument comprised 25 items and conceptualized three factors, viz. trust (10 items), communication (8 items), and alienation (7 items). An example item for trust is “my peers understand me”. For communication example item is “my peers can tell when I am upset about something”, and for alienation it is “my peers don’t understand my problems”. Cronbach’s alpha for the composite factors were, trust = 0.91, communication = 0.91, and alienation = 0.90. Composite reliability for the overall scale was 0.95.

#### Control Variables

In line with prior researchers (e.g., Kling et al., 1999; Twenge & Campbell, 2001), we controlled participants’ gender (coded as male = 1 & female = 2). Further, we controlled the number of siblings in the family as it can significantly impact adolescents’ self-esteem. Although research has found that self-esteem developed steadily across the life span (for a detailed review, see, meta-analysis by Orth et al., 2018), we did not control for the participant’s age, as the survey participants of our study fell within the same age group (between 19–24).

### Analytic Procedure

To test the congruence**/**incongruence, we used polynomial regression coupled with response surface methodology (Edwards & Parry, 1993). We included first-order polynomial terms and higher-order polynomial terms of attachment to parents and peers on self-esteem. To reduce multicollinearity and to facilitate the interpretation of results, we scale-centered the variables (Matta et al., 2015) and then created the second-order polynomial terms. We tested the hypotheses by estimating the following equation.

Self-esteem = β0 + β1(attachment_parents) + β2(attachment_peers) + β3(attachment_parents)2 + β4(attachment_parents x attachment_peers) + β5(attachment_peers)2 + *e*1.

To measure the congruence, we followed the steps given by Edwards and Cable (2009). Accordingly, the first feature of congruence is the curvature along the incongruence line [attachment to parents = attachment to peers (a4), which is calculated as b3 – b4 + b5]. The second feature of congruence includes the first principal axis of the response surface or peak of the response surface. To satisfy the congruence effect, the slope of the first principal axis (p11) should be 1, and the intercept (p10) should be 0 (Edwards & Parry, 1993). In line with Matta et al. (2015), we used 10,000 bootstrapped samples to calculate the 95% bias-corrected confidence intervals for p10 and p11. The third feature is that the slope of the congruence line should be flat. In other words, the level of self-esteem is the same irrespective of whether the attachment to parents and attachment to peers is high or low, a1 (b1 + b2). To assess the asymmetrical incongruence effect, we calculate the slope of the incongruence line, a3 (b1 – b2), and the lateral shift quantity, as they help to specify the magnitude and the direction of the incongruence line, [b2-b1]/[2x (b3-b4+b5)] (Matta et al., 2015). In line with prior researchers who used polynomial regression (e.g., Edwards & Cable, 2009; Matta et al., 2015), we plotted a three-dimensional response surface where the vertical axis corresponds to the values of self-esteem and the perpendicular axis corresponds to the values of attachment to parents and attachment to peers.

### Preliminary Analysis

Before testing our hypotheses, we conducted confirmatory factor analysis (CFA). Attachment to parents and peers was conceptualized as a second-order structure, whereas self-esteem was conceptualized as a first-order structure. We had to add one error-term correlation to the self-esteem and two error-terms to the trust sub-dimension of attachment to parents, two error terms to the communication sub-dimension of attachment to parents. Further, we had to add two error-terms to trust, one error-term to communication, and one error-term to alienation in attachment to peers. After these modifications, all the items loaded on their respective factor significantly with loadings higher than .50. According to the common threshold proposed by Bentler and Bonett (1980) a model has an acceptable good fit if, a) the RMSEA values are ≤ .06 and, b) the CFI and TLI values are ≥ .90, and SRMR values are ≤ .08. The results from CFA indicated that the measurement model fit the data well, as follows: (χ^2^ = 2508.58; *DF* = 1692; *p* < .001; CMIN/*DF* = 1.48; CFI = 0.91; TLI = 0.90; RMSEA = .04; SRMR = .07). We performed Harman's single factor test to examine if common method variance (CMV) was distorting our data (Podsakoff et al., 2003). The results of Harman's single factor test indicated that the first factor accounted for only 27.75% of the variance, which is well below the threshold of 50%.

Mean, standard deviation, and correlation values are presented in Table 1.

**Table 1:** Mean, Standard Deviation, and Inter-Item Correlation Coefficients

Variable		*M*	*SD*	1	2	3	4
1	Gender	1.78	0.41				
2	Siblings	1.57	0.94	.09			
3	Attachment to parents	3.48	0.35	-.03	.27**		
4	Attachment to peers	3.40	0.40	-.03	.11	.42**	
5	Self Esteem	3.50	0.38	-.04	.35**	.61**	.32**

*Note*. *n* = 267.

**p < .01.

## Results

**Figure 2:**
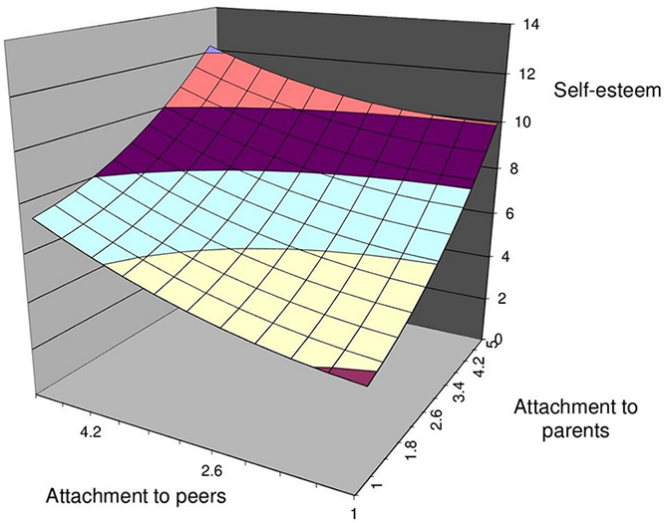
Response Surface for the Relationship Between Attachment to Parents, Attachment to Peers, and Self-Esteem

The polynomial regression results are shown in Table 2, and the resulting surface plots are shown in Figure 2. Hypothesis 1 proposed that more congruence effect of adolescents’ attachment to parents and peers will increase self-esteem. The first feature of congruence is the significant curvature (a4 = b3-b4+b5) of the incongruence line. As shown in Model 2 of Table 2, the curvature of the incongruence line (attachment to parents = -attachment to peers) was not significant (a4 = .45, *p* = .06). Thus, the results of the first feature of the congruence effect do not support it. The second feature of congruence is the slope (p11 = 1.0) and the intercept (p10 = 0) of the first principal axis. The results of bootstrap analysis (10,000) showed that the slope of the principal axis (p11) was not significantly different from 1.0 as the 95% CI included 1.0 [-19.53, 5.32] and an intercept (p10) that was not significantly different from 0 as the 95% CI included 0 [-16.06, 0.15]. Thus, the results provide support for the congruence. The third feature includes the surface along the congruence line (a1, attachment to parents = attachment to peers), which is flat or can vary. The results showed that the slope of the congruence line was significant and positive, a1 [b1+b2] = 0.36, *p* = .004. Although the second and third features support the existence of the congruence effect, the first feature of the congruence effect does not support it. Thus, we reject H1 proposing that adolescents’ self-esteem does not depend on congruence effect of attachment to parents and peers.

**Table 2:** Polynomial Regression Results

Variable	Model 1	Model 2
Constant	3.41**	3.16**
Control variables		
Gender	0.07	-0.03
Siblings	0.14**	0.07
Polynomial Terms		
b1 Attachment to parents		0.34**
b2 Attachment to peers		0.02
b3 Attachment to parents^2^		0.22
b4 Attachment to parents x Attachment to peers		-0.08
b5 Attachment to peers^2^		0.15*
Variance explained		
*R* ^2^	0.13	0.44
Δ*R*^2^	0.13	0.32
Congruence line (Attachment to parents = Attachment to peers)		
Slope (b1 + b2)		0.04**
Curvature (b3 + b4 + b5)		0.03**
Incongruence line (Attachment to parents = -Attachment to peers)		
Slope (b1 - b2)		0.03*
Curvature (b3 - b4 + b5)		0.05
Lateral shift quantity		-0.36

Hypothesis 2 proposes that self-esteem is higher when attachment to parents and peers are at a higher level than when they are at a low level. As shown in Model 2 of Table 2, the slope along the congruence line (attachment to parents = attachment to peers) was positive and significant, a1 [b1+b2] = 0.36, *p* = .004. Moreover, the response surface in Figure 2 shows that self-esteem is higher when both attachments to parents and peers are at a high level. Thus, H2 is supported.

Hypothesis 3 proposes that self-esteem will be higher when attachment to parents is higher than attachment to peers. First, we examined the slope along the incongruence line (attachment to parents = -attachment to peers), and the results showed that self-esteem was higher when attachment to parents was higher than attachment to peers (a3, b1–b2 = 0.33, *p* = .047). To further examine the direction and magnitude of the incongruence effect, we tested for the lateral shift quantity. Providing additional evidence to support H3, the results of lateral shift quantity were negative and significant, β = -0.36; 95% [CI = -3.84, -0.02]. Our results also consisted of the response surface in Figure 2, which shows self-esteem was higher when the attachment to parents was high. Thus, H3 is supported.

## Discussion

Based on attachment theory, this study aimed to examine the effect of (in)congruence of adolescents’ attachment to parents and peers on self-esteem. In Hypothesis 1, we postulated that the congruence of attachment to parents and peers increase adolescents’ self-esteem. However, the results of polynomial regression analysis show that the congruence effect of attachment to parents and peers on self-esteem is not significant. Attachment theory highlights that individuals may move from one attachment figure to another if they feel that the current attachment figure is not available or is insecure (Ainsworth, 1989; Bowlby, 1969, 1982). For example, if a married person feels the partner is not trustworthy, he/ she may get a divorce and seek a new relationship. Likewise, individuals often have a strong attachment to one figure while maintaining a loose attachment with others. Thus, we believe adolescents form a strong emotional bond with parties who are close to them. Therefore, the results of this study contribute to the attachment theory by demonstrating that the simultaneous effect of parental and peer attachment is not important for developing one's feeling of self-worth.

In fact, the findings of this study support our argument of self-esteem of adolescents is high when both attachment to parents and attachment to peers is at a high level than at a low level (Hypothesis 2). Thus, the quality of attachments seems to be vital in strengthening adolescents’ self-esteem. This finding is consistent with previous studies such as Gorrese and Ruggieri (2013) and Wilkinson (2004), which have shown the positive association between high-quality attachment and self-worth.

Moreover, the results support our Hypothesis 3 that adolescents’ manifest high self-esteem when their attachment to parents is higher than their attachment to peers. The relationships that adolescents build with their friends and romantic partners are less stable than the bond they build up with their peers (Berndt, 1982). According to the attachment theory (Bowlby, 1969), proximity to the attachment figure is one of the main requisites of strong emotional bonds. We believe that adolescents live in close proximity to their parents than their peers. Especially in Asian countries, the family is the primary unit of society, and adolescents normally live with their parents. Thus, close attachment to parents can be the most influential predictor of the likelihood of adolescents developing their self-esteem. Further, these findings are consistent with those of prior research (e.g., de Jonge et al., 2022; Gorrese & Ruggieri, 2013; Mattanah et al., 2011), suggesting that parents play a more important role than peers in developing adolescents’ self-esteem.

This study provides several theoretical implications. First, the findings of this study contribute to the attachment theory by examining the joint effect of adolescents’ attachment to parents and peers on their self-esteem. Although previous studies have examined the effect of social relationships on self-esteem, most of these studies (e.g., Harris & Orth, 2020; Marshall et al., 2015) generally ignored their simultaneous or joint effect. However, adolescents do interact with their parents and peers simultaneously. Therefore, the present study provides a clear picture of social interactions and helps to enrich the current understanding of adolescents’ attachment to parents and peers on self-esteem. Second, this study provides an answer to the question of “what is the impact of peer attachment on self-esteem?” The information found in the literature on the association between peer attachment and self-esteem is inconsistent. In line with previous researchers such as Armsden and Greenberg (1987), and Gorrese and Ruggieri (2013), our study shows attachment to peers is important for an adolescent’s self-esteem development; however, attachment to parents is much more important. Thus, this study contributes to clarifying the inconsistencies in the literature. Third, in our study, we examined the association between attachment to parents and peers and adolescents’ self-esteem based on a sample from Sri Lanka. As most of the extant studies relied on Western countries, there is a knowledge gap about this topic in the non-western countries. Therefore, this study contributes to the existing literature by increasing our understanding of adolescents’ self-esteem at a global level.

In addition, the results of this study inform the policymakers on how to encourage parents to continue their engagement with children even into their adolescent years as attachment to parents can significantly improve their offspring’s self-esteem. Therefore, parents should spend more time with their children and provide them emotional support whenever they are in need. Moreover, parents need to instill the need for self-improvement and praise their children’s efforts even when they fall short of their goals.

### Limitations and Directions for Future Researchers

However, the study does have its limitations. First, although we controlled the participant’s gender, we did not test how these relationships vary between the genders. Prior research has shown gender differences in self-esteem (e.g., Gorrese & Ruggieri, 2013; Twenge & Campbell, 2001). Thus, we recommend that future researchers test the gender effect on attachment and self-esteem. Attachment theory has conceptualized three main dimensions of attachment (attachment styles) as secure, avoidance, and anxiety (Ainsworth et al., 1978). An individual’s attachment style influences the nature of the emotional bond with the attachment figure (Ainsworth et al., 1978). Thus, the results of this study may vary with individuals who have different attachment styles. Therefore, we recommend that future researchers test the model by integrating the attachment styles. It must also be mentioned that data collection for this study was limited to one point in time, and data were collected only from adolescents age between 19–24. Self-appraisal of emotional connection (attachment to parents and peers) may not present a complete picture of one’s social relationships. Therefore, the results of this study might be influenced by common method bias. Hence, future researchers are advised to collect data from multiple sources (e.g., parents and peers) and at different time points to improve the robustness of the study design. Moreover, as this study’s sample consisted with adolescents aged from 19–24 (who are at the age of end of their adolescence), we recommend further researchers to test over model over other ages too.

### Conclusion

The aim of this study was to examine the simultaneous effects of adolescents’ attachment to parents and peers on self-esteem. The results of polynomial regression analysis suggest that the relationships that adolescents establish with their parents are more significant than the relationship that they establish with their peers to develop their self-esteem. Specifically, the emotional bond that they establish with their parents will foster high self-esteem. Thus, the development of strong emotional bonds with parents allows adolescents to build high self-esteem, which will eventually lead to effectively dealing with negative life experiences such as stress and low self-confidence. Overall, by elucidating the (in)congruence effect of adolescents’ attachment to parents and peers on self-esteem, the findings of this study help us to gain a better understanding of how the support of parents and peers can improve an adolescent’s self-esteem.

## Supplementary Materials

The supplementary materials provided are the data sets, data analyses, research instrument, and research results that support the findings of this study (for access see Index of Supplementary Materials below).



KarunarathneR. A. I. C.
 (2023). Supplementary materials to "Parents or peers? (In)congruence effect of adolescents’ attachment to parents and peers on self-esteem"
[Research data]. PsychOpen. 10.23668/psycharchives.12875
PMC1050820537731895

KarunarathneR. A. I. C.
 (2023). Supplementary materials to "Parents or peers? (In)congruence effect of adolescents’ attachment to parents and peers on self-esteem"
[Analyses, instruments]. PsychOpen. 10.23668/psycharchives.12876
PMC1050820537731895

## Data Availability

Data are freely available, see Karunarathne (2023).
